# Gestational Diabetes Mellitus in the Setting of Polycystic Ovarian Syndrome: A Systematic Review

**DOI:** 10.7759/cureus.50725

**Published:** 2023-12-18

**Authors:** Ethan Slouha, Vanessa C. Alvarez, Kaitlyn M Gates, Nana Mansa N Ankrah, Lucy A Clunes, Theofanis F Kollias

**Affiliations:** 1 Anatomical Sciences, St. George's University School of Medicine, St. George's, GRD; 2 Pharmacology, St. George's University School of Medicine, St. George's, GRD; 3 Medicine, St. George's University School of Medicine, St. George's, GRD; 4 Pharmacology, St. George's University, St. George's, GRD; 5 Microbiology, Immunology, and Pharmacology, St. George's University School of Medicine, St. George's, GRD

**Keywords:** pcos, gestational diabetes mellitus, pregnancy, endocrinology, obstetrics, polycystic ovarian syndrome, gestational diabetes

## Abstract

Gestational diabetes mellitus (GDM) is the most common complication of pregnancy that arises in the 2nd and 3rd trimesters, leading to significant complications for the mother and her neonates, such as an increased rate of pregnancy-induced hypertension and miscarriages, while neonates may have a large birth weight, hypoglycemia, or macrosomnia. Numerous risk factors can lead to GDM; however, a significant one is polycystic ovarian syndrome (PCOS). PCOS is the most common endocrine pathology beginning before puberty, and due to significant hormonal changes, it is not diagnosed until after puberty. PCOS requires at least three of the following symptoms: hyperandrogenism, menstrual irregularities, or polycystic ovary morphology. While it is agreed that women with PCOS are at a significantly increased risk of GDM, no publication to our knowledge has evaluated the full relationship of GDM in the setting of PCOS. This paper aimed to assess this relationship and determine how it may differ for pregnant women with only GDM by determining the prevalence of GDM, the variations within phenotypes, the influence of fertilization methods, specific risk factors, maternal outcomes, and neonatal outcomes. The prevalence of GDM was significantly increased in women with PCOS compared to healthy controls, and some studies have found that phenotype A may be more likely to lead to GDM. Risk factors were similar to pregnant women with only GDM, but with GDM and PCOS specifically, preconception low sex hormone-binding globulin, increased BMI > 25 kg/m2, and preconception impaired glucose tolerance were specific. While maternal outcomes were similar to pregnant women with only GDM, women with GDM and PCOS were even more likely to develop pregnancy-induced hypertension and early miscarriage. Neonates from mothers with GDM and PCOS were more likely to have low birth weights compared to mothers with just GDM who had high birth weights. The evaluation of the relationship between GDM and PCOS allows for illumination of the need to evaluate influences that currently lack research, such as phenotype variation and influences of fertilization method. This also promotes the need to develop predictive algorithms based on risk factors to prevent these adverse outcomes for mothers and neonates.

## Introduction and background

Gestational diabetes mellitus

Pregnancy is generally associated with increased insulin resistance due to lactogen secretion, growth hormone, tumor necrosis factor-alpha, estrogen, and progesterone [1]. The definition of gestational diabetes mellitus (GDM) is any grade of glucose intolerance at onset or first recognition in pregnancy and is classified by responsiveness to nutritional therapy (A1GDM) or requiring medication (A2GDM) [1,2]. GDM is the most common medical complication of pregnancy and typically develops in the second and third trimesters of pregnancy, affecting between 2% and 10% of pregnancies in the United States [1,2]. Screening includes evaluation of patient history, family history of type 2 diabetes mellitus, oral glucose tolerance test, and past medical obstetric outcomes [2]. Screening occurs at 24-28 weeks with a 50-g, one-hour oral glucose tolerance test greater than 130 mL/dL [1,2].

Two possible etiologies have been identified: pancreatic b-cell dysfunction or the hindered response to glycemic levels [1-3]. Hindered response to glycemic levels is caused by significant insulin resistance due to hormonal releases such as lactogen, growth hormone, prolactin, and progesterone [1,2]. Lactogen is released to induce metabolic changes and support preserving fetal nutritional status [2]. Lactogen leads to variations and modifications at insulin receptors, reduced tyrosine kinase phosphorylation, and remodeling substrate-1 and phosphatidylinositol 3-kinase [2]. GDM can be managed without medication with nutritional therapy or through medicines like insulin and metformin that achieve optimal glycemic control [1,2].

Maternal complications of GDM include preeclampsia, increased risk of cesarean delivery, and increased risk of developing type 2 diabetes mellitus [1,2]. Fetal complications can occur, such as macrosomia, polycythemia, fetal hyperglycemia, neonatal hypoglycemia, shoulder dystocia, neonatal respiratory distress syndrome, and increased perinatal mortality [1,2]. Women should be educated to self-monitor blood glucose levels up to four times a day, fasting, and one to two hours post meal to reduce the risk of GDM [1]. Following delivery, fasting blood glucose is monitored for 24-72 hours and six weeks later to determine if the mother’s hyperglycemic control is back to normal, and the oral glucose tolerance test is repeated every three years [1]. Clinical risk factors include increased body weight, older age, reduced physical activity, cardiovascular disease, previous history, and PCOS [1,2].

Polycystic ovarian syndrome

Polycystic ovarian syndrome (PCOS) is a multifactorial disorder that is the most common endocrine pathology in females of reproductive age [4-6]. Symptoms of PCOS begin during early pubertal years but can be challenging to diagnose due to the effects of puberty [4,6]. It was initially discovered in 1935, and the prevalence now ranges from 5% to 15%, depending on what diagnostic criteria clinicians use [4]. PCOS is a diagnosis of exclusion as many disorders, such as thyroid disease, non-classical congenital adrenal hyperplasia, and hyperprolactinemia, mimic the clinical features [4,5]. It cannot be diagnosed with standard diagnostic tests like biopsy, blood test, and culture [5]. PCOS, according to the Rotterdam criteria, is characterized by having two or more of the following: hyperandrogenism, irregular menstrual periods, and polycystic ovaries (PCO) [4-6]. PCOS also consists of a variety of symptoms, with the most common being hirsutism, PCO, alopecia, irregular periods, and infertility [4]. PCOS has also recently been divided into four phenotypes: A - hyperandrogenism, ovulatory dysfunction, and PCO morphology; B - hyperandrogenism and ovulatory dysfunction; C - hyperandrogenism and PCO morphology; D - ovulatory dysfunction and PCO morphology [6].

While the prevalence of PCOS is significant, it is widely underdiagnosed and usually requires more than one visit or visiting multiple physicians to be identified, typically in a one-year time frame [4]. There are numerous comorbidities, such as infertility, obesity, type 2 diabetes mellitus, metabolic syndrome, impaired glucose tolerance, and depression in PCOS, but this list is not exhaustive [4,5]. Almost all of the causes of PCOS are associated with functional ovarian hyperandrogenism, characterized by dysregulation in the secretion of androgen [4,5]. Genes involving various points of steroidogenesis and androgenic pathways like LHCGR and EPHX1 have been identified to lead to PCOS, as twin studies show up to 70% heritability [4,5]. Environmental factors such as obesity, bisphenol A toxin, and insulin resistance were found to influence the activation of these genes significantly [4,5].

First-line treatment involves lifestyle modification, such as calorie-restrictive diets and exercise, to address associated weight loss, hirsutism, regulation of the menstrual cycle, and impaired glucose tolerance [4,5]. Hormonal contraceptives like oral contraceptives, patches, or vaginal rings are effective against the symptoms of PCOS [4,5]. The progestin found in hormonal contraceptives decreases luteinizing hormone (LH) levels, indirectly decreasing ovarian androgen production and increasing sex hormone-binding globulin (SHBG) [4]. Women with PCOS often have infertility, which can be treated with clomiphene citrate, which is a selective estrogen receptor modular and competitive inhibitor of estrogen receptors [4]. Infertility can also be treated with assisted reproductive technology (ART), such as in vitro fertilization (IVF) and intracytoplasmic sperm injection (ICSI) when hormonal treatment fails [7,8].

Aim

Women with PCOS are considered a high-risk population that continues during pregnancy and are at a significantly increased risk of developing GDM. GDM has been shown to have significant effects on maternal and neonatal outcomes, typically affecting delivery time, birth weight, and neonatal health status. GDM risk can potentially be predicted and possibly prevented with predictor evaluation, but ongoing research is still trying to assess this possibility. However, it is essential to fully understand the association between PCOS and GDM, more than just the known increased risk. This paper aims to evaluate the prevalence of GDM with the variations in PCOS phenotypes, influences of fertilization methods, associated risk factors, and maternal and neonatal outcomes. We plan to evaluate the whole relationship and the impact of coexistence to highlight where research may be lacking and what may offer further insight into the development of GDM.

## Review

Methods

The present systematic review was performed with strict adherence to the Preferred Reporting Items for Systematic Reviews and Meta-Analyses (PRISMA) guidelines. This resulted in a methodical and calculated search of the current literature found in ScienceDirect, ProQuest, and PubMed between January 1, 2013, and November 1, 2023. The keywords used to conduct the search were "polycystic ovarian syndrome and gestational diabetes mellitus" and were chosen to acquire publications that covered an array of subtopics within this theme. The investigation was arranged around peer-reviewed experimental and observational publications. Publications in languages other than English, studies that were duplicates, and studies published before 2013 were excluded. After obtaining the publications after automatic screening, they were evaluated manually based on their title, abstract, study, and full-text availability. The preliminary investigation of the catalogs used resulted in 10,694 publications. The title and abstracts of the acquired publications were cross-references with the keywords and chosen subtopics, allowing for the publication list to be narrowed down and aligned with the aim of this review. A total of 50 publications were obtained according to the criteria mentioned below.

Inclusion Criteria

The publications were selected based on the following criteria: studies focusing on GDM in the presence of PCOS, peer-reviewed experimental or observational studies, studies performed on humans, full-text availability, and publications between 2013 and 2023.

Exclusion Criteria

**Figure 1:**
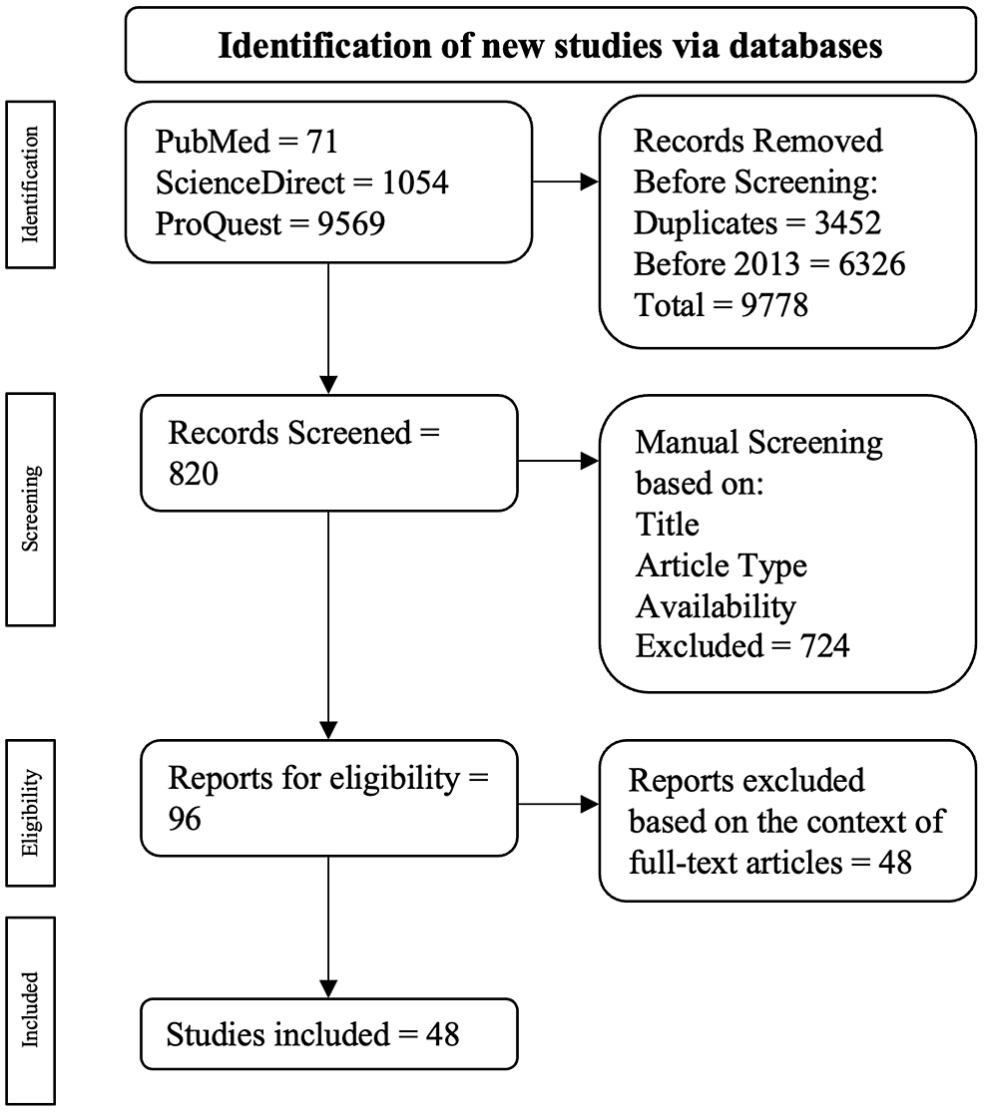
Algorithm employed based on the inclusion and exclusion criteria. The flowchart was adapted to the PRISMA guidelines. The flowchart was adapted to the Preferred Reporting Items for Systematic Reviews and Meta-Analyses (PRISMA) guidelines [9].

The publications excluded from this review were based on the following criteria: articles written in languages other than English, duplications, and no full-text availability. The procurement algorithm using the stated inclusion and exclusion criteria is drawn out in Figure 1.

Bias

All publications procured were examined for bias through the GRADE (grading of recommendation, development, and evaluation) scale and were graded with a moderate bias rating.

Results

A total of 10,694 publications were populated: 71 were from PubMed, 1054 were from ScienceDirect, and 9569 were from ProQuest. Among the exclusions, 3452 were duplicate publications, and 6326 were published before 2013. This resulted in 9778 publications being excluded throughout the automatic screening algorithm, leading to 820 publications for manual screening. Publications were then examined manually based on their title, study, and full-text availability, resulting in 96 publications being chosen for eligibility for full-text examination. Ultimately, 48 publications were selected.

PCOS is a significant risk factor for developing GDM, and pregnant women with PCOS have a significantly increased prevalence of GDM. Pregnant women with GDM in the setting of PCOS were also discovered to have significantly increased BMI compared to controls with just GDM. Concerning PCOS phenotypes, phenotype A was more likely to increase GDM prevalence; however, when comparing all studies, this was insignificant. The influence of fertilization methods for women with PCOS was inconsistent, with some studies reporting that ART tends to reduce the prevalence of GDM. Still, IVF specifically increases the prevalence of GDM. There is a wide range of risk factors that lead to the development of GDM in the setting of PCOS, and while there is overlap with the development of GDM in healthy controls, low SHBG preconception was found to be a significant risk factor, along with BMI and preconception impaired glucose tolerance. Concerning maternal complications, there was a significantly increased risk of developing pregnancy-induced hypertension with a combination of GDM and PCOS, and there were some studies that found an increased rate of early miscarriage. Neonates were more likely to have low birth weight in mothers having GDM and PCOS, which is different from mothers solely having GDM as they were more likely to have neonates with high birth weights and macrosomnia.

Discussion

Risk Factors That Increase the Incidence and Prevalence of Gestational Diabetes Mellitus

The development of GDM in pregnant women with PCOS is associated with numerous risk factors. Irregular menstrual patterns are a significant and independent risk factor for the development of GDM [10-12]. The most common risk factor identified across the majority of studies is a high BMI of over 25 kg/m2 [12-25]. Other associated risk factors include increased fast blood glucose, free androgen index, insulin resistance, cholesterol, blood pressure, testosterone concentrations, age, fasting insulin, HbA1c, presence of metabolic syndrome, short stature, lower high-density lipoprotein, and preconception impaired glucose tolerance [14,17,19-22,24,26-29]. Another predictor of GDM is SHBG, which is shown to have a significantly negative association [19,21,25]. Mumm et al., however, observed no difference in SHBG. Female fetal sex was also found to be a statistically significant risk factor for developing GDM by almost twofold [30]. Having a greater first-degree family history of type 2 diabetes mellitus, family history of GDM, prior preterm birth, and early pregnancy loss can also contribute to the development of GDM [19,20,22,31]. Ashrafi et al. found that a significant number of patients with PCOS who subsequently developed GDM had a history of hypothyroidism, suggesting it is a risk factor for, or at least a strong predictor of, developing GDM [10].

Maternal Complications Associated With Gestational Diabetes Mellitus

With GDM being present in the setting of PCOS, the question is whether or not there is an increased risk or presence of other pregnancy complications. Several recent studies observed that PCOS with GDM was found to be a significant risk factor and increases the incidence of pregnancy-induced hypertension compared to healthy controls with just GDM [27,32-36]. One study, however, found no significant increase in the incidence of pregnancy-induced hypertension [37]. GDM with PCOS has also been found to increase the incidence of polyhydramnios, preterm premature rupture of membranes, and moderate or severe ovarian hyperstimulation syndrome [27,35,36]. Being diagnosed with GDM in the 1st trimester in women with PCOS was also non-significantly associated with an increased rate of late miscarriage and a lower rate of live births [16,29]. Yu et al., however, found that the overall incidence of abortion, spontaneous preterm birth, GDM, a hypertensive disorder in pregnancy, premature rupture of membranes, and macrosomia were significantly higher in the amenorrheic group compared to oligomenorrhea and regular menstrual cycles [12].

Neonatal Outcomes With Gestational Diabetes Mellitus

Just like with additional maternal complications, the combination of GDM and PCOS must also lead to additional neonatal complications. The majority of studies found that neonates born to women with both PCOS and GDM were more likely to have fetal growth restrictions, leading to lower birthweight compared to neonates from mothers with just GDM [12,16,17,35,38-41]. A few studies, however, showed an increase in birthweight and macrosomnia [29,36,42]. Amenorrheic menstrual cycles specifically were statistically correlated with macrosomnia in one study, though [12]. Helseth et al. observed that within their 1st year of life, infants born to patients with PCOS and GDM exhibited less weight gain than control, but the difference was only approaching significance [17].

Neonatal hypoglycemia developed in 17% of infants born to patients with PCOS and GDM showed that the presence of PCOS with GDM, compared to GDM alone, is associated with a 3.2-fold increase in the risk of neonatal hypoglycemia development [32]. Among patients with PCOS, the odds of developing neonatal jaundice and respiratory complications were significantly higher compared to the non-PCOS control group [43]. ICU admissions differed among the three PCOS groups; however, after correction, this was not statistically significant [44]. Among the four phenotypes of PCOS studied, no statistically significant differences were found in the incidence of neonatal birth weight, neonatal icterus, NICU admission, or neonatal death [45]. While a good amount of studies found additional risks, several studies found no difference in Apgar, birth weight, and incidence of hypoglycemia, which was also seen in comparing fertilization methods [37,39,43,44,46].

Incidence of Gestational Diabetes Mellitus in Polycystic Ovarian Syndrome

PCOS is positively associated with and leads to an increased risk of developing GDM in pregnant women [14,24,26,34-36,39,41,42,46-51]. One study found that there was a 2.7-fold increased risk of the development of GDM in pregnant women with PCOS [11]. The current estimated prevalence of GDM in pregnant women with PCOS between studies was 26%, significantly higher than healthy controls [10,16,19,21-23,26,27,35,38,40,41,44,47,50-55]. However, several studies observed that there was no significant difference in the incidence or prevalence of GDM between pregnant women with and without PCOS [13,15,31,52,56]. When comparing whether or not women were amenorrheic or oligomenorrheic before pregnancy, there were conflicting results where one study found that the amenorrheic group was significantly more likely to develop GDM by 7.69%, while the other study found that oligomenorrhea was significantly associated with GDM concerning women with PCOS [12,14]. Classically, women with PCOS tend to be overweight. This trend continued during pregnancy when GDM was diagnosed, with most studies observing that pregnant women with PCOS had a significantly higher BMI [13,18,23,33,43]. Another interesting finding is that the implantation of an oocyte from a woman with PCOS into a woman without PCOS does not alter the baseline prevalence of GDM [46].

Phenotype Variation With the Prevalence and Incidence of Gestation Diabetes Mellitus

PCOS consists of four phenotypes A-D, but in general, it has been observed that pregnant women with PCOS consisting of hyperandrogenism and oligomenorrhea, such as A and B, were at an increased risk of developing GDM [47,48]. Conclusions of which phenotype was more likely to develop GDM were split, with some studies observing that phenotype A was associated with the highest incidence of GDM with up to 27.5% of patients [26,28,44,45,53,57]. In contrast, other studies found no significant difference [26,28,44,45,53,57]. One study, however, determined that phenotype D was associated with the lowest incidence of GDM [45]. Fasting blood glucose was also significantly higher in phenotype A than in D [45]. Despite phenotype A possibly being associated with the highest risk of GDM, one study observed that fasting blood glucose was significantly higher in phenotype B, confirmed by glucose monitoring at three-hour oral glucose tolerance test (OGTT) [57].

Fertilization for Women With PCOS and Its Effects on Gestational Diabetes Mellitus

Women who suffer from PCOS may need to undergo assistance for fertilization to occur, and this can be through hormone regulation or IVF. Women with PCOS who underwent ovulation induction using clomiphene citrate and follicle-stimulating hormone (FSH) have a significant increase in the incidence of GDM developing during pregnancy [14]. There was an increased probability of GDM among PCOS patients who underwent the artificial cycle method compared to those who completed the natural cycle method, but this was insignificant [39]. Some studies observed that there was a decreased risk of developing GDM in women with PCOS who underwent ART, but this did not correlate with what studies have found about IVF and ICSI [41,42]. Two studies found that there was a positive correlation between GDM development in women with PCOS who underwent IVF and ICSI [14,39]. Zhang et al. went further into analyzing fertilization methods and the development of GDM and observed no difference in the incidence when comparing fresh and frozen embryos [25]. One study, however, found that the use of ART did not significantly alter the incidence of GDM in women with PCOS [33].

The articles synthesized to compose this review can be found in Table 1.

**Table 1:** Summary of articles per the PRISMA guidelines. PCOS: polycystic ovarian syndrome; GDM: gestational diabetes mellitus; PIH: pregnancy-induced hypertension; PCO: polycystic ovary; IVF: in vitro fertilization; ICSI: intracytoplasmic sperm injection; SHBG: sex hormone-binding globulin; MetS: metabolic syndrome; WHO: World Health Organization; T2DM: type 2 diabetes mellitus; PRISMA: Preferred Reporting Items for Systematic Reviews and Meta-Analyses; ART: assisted reproductive technology; HbA1c: glycosylated hemoglobin.

	Author	Country	Design & study population	Findings	Conclusion
1	Wang et al., 2013 [41]	China	Cohort study (n = 338)	Regardless of the method of conception, the incidence of GDM was higher in women with PCOS compared to women without. PCOS was also associated with PIH, preterm delivery, and fetal growth restriction.	PCOS is an independent risk factor for developing GDM.
2	Foroozanfard et al., 2020 [26]	Iran	Prospective cohort study (n = 90)	Patients with PCOS consisting of hyperandrogenism, menstrual dysfunction, and PCO and a significantly higher incidence of GDM compared to controls. High androgen levels were the significantly strongest predictor of developing GDM during pregnancy.	PCOS with hyperandrogenism, menstrual dysfunctions, and polycystic ovaries is significantly correlated with GDM compared to healthy women and other phenotypes of PCOS patients.
3	Appiah et al., 2023 [47]	USA	Longitudinal observational study (n = 455)	In the absence of PCOS, oligomenorrhea and hyperandrogenism are not associated with GDM. However, an elevated risk of GDM was found among women with PCOS. Total testosterone and free testosterone levels were not significantly associated with GDM.	A positive association was found between PCOS and GDM.
4	Ashrafi et al., 2014 [10]	Iran	Cross-sectional study (n = 702)	The incidence of GDM was significant for patients with PCOS at 44.4%, with women who have PCOS undergoing ART at 29.9%, and for non-PCOS with spontaneous conceptions at 7.9%.	Women with PCOS and a history of infertility are at greater risk of developing GDM.
5	Ashrafi et al., 2017 [57].	Iran	Cross-sectional study (n = 208)	Increased prevalence of GDM was found in phenotype A, then C, B, and D, but the relationship is not statistically significant. Triglycerides were significantly greater in PCOS phenotype B.	PCOS phenotypes A and B were found to have an increase in metabolic disorders.
6	Yu et al., 2021 [12]	China	Retrospective cohort study (n = 1,834)	In patients with PCOS, amenorrhea was associated with a statistically significant higher incidence of GDM and macrosomia compared to patients with PCOS who had regular menstrual cycles or oligomenorrhea. Amenorrhea was also found to be associated with higher pregnancy complications.	Amenorrhea was an independent risk factor associated with adverse pregnancy outcomes in patients with PCOS who underwent IVF/ICSI.
7	Odsæter et al., 2015 [40]	Norway	Prospective cohort study (n = 228)	There is no statistically significant association between HbA1c and diagnosing GDM in the first trimester. However, HbA1c during the first trimester was statistically significantly associated with preeclampsia.	HbA1c cannot be used to exclude or predict GDM during the first trimester in women with PCOS. However, HbA1c may be more useful in predicting preeclampsia in PCOS than a GDM diagnosis alone.
8	Pattnaik et al., 2022 [54]	India	Prospective case-control study (n = 102)	High BMI was twice as common in PCOS patients than in controls. PCOS with pregnancy complications was associated with a BMI of 25 to 29.9 kg/m^2^. Reproductive assistance technology was needed in 86.3% of PCOS patients, and 17.6% developed GDM.	Pregnant women with PCOS are more likely to encounter complications such as spontaneous abortions, GDM, and preterm birth.
9	Yang et al., 2023 [51]	Korea	Retrospective cohort study (n = 73,281)	GDM and pregnancy-induced hypertension were higher in the PCOS group than in the control group. An adjusted analysis concluded that there was an increased risk of GDM among women with PCOS.	PCOS is associated with an increased risk of developing GDM.
10	Ni et al., 2022 [39]	China	Retrospective cohort study (n = 15,990)	PCOS patients were more likely to have an increase in the prevalence of GDM. They also had an increased risk of neonatal complications.	Regardless of fertilization methods, PCOS patients continue to have an elevated risk for GDM during pregnancy.
11	Mills et al., 2020 [49]	United States	Retrospective cohort study (n = 9,096,788)	Women with PCOS were found to have a two-fold increased risk for developing GDM, independent of obesity before pregnancy. PCOS patients are 2.3 times more likely to develop GDM with multiple gestations than non-PCOS patients.	PCOS is, therefore, a strong, independent risk factor for women developing PCOS as compared to non-PCOS patients.
12	Jiang et al., 2022 [46]	China	Retrospective cohort study (n = 62)	Pregnancy complications, including GDM, were not significantly different in single vitrified-warmed blastocyst transfer between the PCOS and non-PCOS groups. No differences between the PCOS and non-PCOS groups were reported in pregnancy outcomes and offspring follow-ups.	PCOS donor oocyte does not affect pregnancy outcomes and offspring follow-up.
13	Bahri Khomami et al., 2019 [38]	New Zealand	Prospective cohort study (n = 5,628)	PCOS is associated with a higher risk of developing GDM. When adjusted for lifestyle factors, this association was no longer significant compared to women without PCOS. PCOS was significantly associated with a decreased risk of large for gestational-age infants.	PCOS is associated with a decreased risk of large for gestational-age infants but shows no significant relationship with GDM when adjusted for lifestyle factors.
14	Kollmann et al., 2015 [44]	Austria	Retrospective cohort study (n = 885)	Patients with PCOS, compared to those without, had an increased risk for maternal complications, including GDM. Additionally, there was no difference in neonatal outcomes between women with and without PCOS.	There is an increased risk of developing maternal complications, including GDM, in women with PCOS compared to women without PCOS.
15	Abdulkhalikova et al., 2021 [13]	Slovenia	Case-control study (n = 269)	PCOS patients tended to be overweight/obese. Neonates with macrosomia were more common in PCOS. BMI was a significant risk for the development of GDM in patients with PCOS.	GDM did not differ between patients with and without PCOS, however, GDM did differ for patients with PCOS who were normal or overweight.
16	Sassin et al., 2023 [30]	United States	Prospective cohort study (n = 7,531)	Pregnant women with PCOS and a history of infertility are at significantly greater risk of developing GDM if the fetus is of female sex.	Women with PCOS and infertility have a significant risk of developing GDM if carrying a female fetus.
17	Zhang et al., 2016 [25]	China	Cross-sectional study (n = 268)	In pregnant Chinese women with PCOS, pre-pregnancy waist-to-hip ratio, SHBG, insulin resistance, and gestation weight gain before 24 weeks were found to be strong risk factors and predictors of GDM development.	Waist-to-hip ratio, sex-hormone binding globulin, insulin resistance, and gestation weight gain before 24 weeks were significantly associated with GDM in Chinese women with PCOS.
18	Liu et al., 2020 [52]	China	Retrospective cohort study (n = 7,678)	Having PCOS is associated with an increased risk of developing adverse pregnancy outcomes but is not statistically associated with an increased risk of GDM.	Women with PCOS are at an increased risk of adverse pregnancy outcomes.
19	Pan et al., 2015 [50]	Taiwan	Prospective cohort study (n = 34,199)	Women with a history of PCOS develop GDM more frequently than women without a history of PCOS. Taking medication for PCOS or using oral anti-hypoglycemic medications before becoming pregnant does not reduce the risk of developing GDM in women with PCOS.	PCOS is associated with an increased risk of developing GDM.
20	Ouyang et al., 2023 [22]	China	Retrospective cohort study (n = 434)	Serum levels of HbA1c, age, total cholesterol, low-density lipoprotein, systolic blood pressure, family history, BMI, and testosterone during the first trimester were predictive factors in the development of GDM in the second trimester among gravid PCOS patients.	Independent risk factors for the development of GDM in gravid women with PCOS include total cholesterol, age, HbA1c, BMI, and family history.
21	Mehrabian et al., 2013 [21]	Iran	Prospective cohort study (n = 180)	27.8% of pregnant women with PCOS developed GDM. This was associated with lower levels of SHBG before conception.	Low SHBG before conception increases the risk of GDM in pregnant women with PCOS.
22	Sterling et al., 2016 [36]	Canada	Retrospective cohort study (n = 394)	Women with PCOS are at an increased risk of developing GDM and hypertensive disorders of pregnancy. PCOS is also associated with large gestational-age infants.	PCOS is a risk factor for GDM, hypertensive disorders of pregnancy, and large for gestational age infants.
23	West et al., 2020 [24]	Finland	Cohort study (n = 1,579)	PCOS, either clinically diagnosed or self-reported, is not an independent risk factor for developing GDM. Hyperandrogenism at 31 years of age was significantly associated with the development of GDM.	PCOS is not a risk factor for GDM. Hyperandrogenism and obesity are risk factors for developing GDM.
24	Xia et al., 2017 [55]	China	Prospective cohort study (n = 94)	In women diagnosed with PCOS, preconception insulin under the curve and SHBG were significant risk factors in the development of GDM.	Lower sex hormone-binding globulin and insulin under the curve were associated with GDM in patients previously diagnosed with PCOS.
25	Si et al., 2023 [28]	China	Retrospective cohort study (n = 1,313)	Increased insulin resistance and MetS were found among those with hyperandrogenism and overweight, leading to an increased risk of GDM.	MetS was found to be a significant risk factor for cumulative live birth, preterm birth, and GDM in PCOS patients receiving IVF.
26	Fougner et al., 2021 [16]	Norway	Cohort study (n = 722)	Diagnosis of GDM in the 1^st^ trimester was associated with an increased risk for late miscarriage. When using the WHO 2013 GDM criteria, the usage of metformin decreased the risk of late miscarriages and preterm births in women with GDM.	GDM did not increase the risk for pregnancy complications other than late miscarriage for those diagnosed with GDM in the 1^st^ trimester.
27	Wei et al., 2017 [29]	China	Secondary analysis (n = 1,429)	In women with PCOS, preconception-impaired glucose tolerance leads to an increased risk of GDM in both singleton and twin pregnancies and pregnancy loss in singleton pregnancies. Preconception-impaired glucose tolerance leads to a higher risk for large gestational-age neonates compared to women with PCOS with normoglycemia.	Preconception-impaired glucose tolerance leads to an increased risk of developing GDM in women with PCOS as well as pregnancy loss in singleton pregnancies, and large for gestational age neonates.
28	Feichtinger et al., 2020 [15]	Austria	Prospective cohort study (n = 67)	PCOS was not a risk factor for the development of GDM. Having a higher body weight was significantly associated with insulin resistance in pregnancy.	BMI is a more significant risk factor than having PCOS for the development of GDM.
29	Mustaniemi et al., 2018 [31]	Finland	Case-control study (n = 1,941)	PCOS is not an independent risk factor for developing GDM. Risk factors include obesity, age 35 or higher, pregnant woman’s mother previously having GDM, pregnant woman having a parent with T2DM, and pregnant woman having a previous preterm birth.	Obesity and advanced maternal age are independent risk factors for the development of GDM. PCOS is not a risk factor for developing GDM.
30	Hu et al., 2021 [56]	China	Retrospective cohort study (n = 2,121)	Patients with PCOS and without hyperandrogenism had higher rates of cumulative live births and clinical pregnancy. There was no difference between rates of preterm birth, GDM, hypertensive disorders of pregnancy, fetal malformation, placenta previa, low birth weight, and macrosomia between groups.	Patients with PCOS but without hyperandrogenism have no real differences in pregnancy outcomes compared to healthy controls.
31	Qiu et al., 2022 [35]	China	Retrospective cohort study (n = 14,630)	Women with PCOS were more likely to develop GDM, PIH, and preterm premature rupture of membranes compared to women without PCOS. It was also shown that PCOS women who underwent menstrual cycle stimulation, as opposed to hormone replacement therapy, had a lower incidence of GDM.	PCOS is an independent risk factor for the development of GDM and is associated with PIH as well in women who underwent frozen-thawed embryo transfers.
32	Mumm et al., 2015 [53]	Denmark	Cohort study (n = 1,194)	There was no statistical difference in the prevalence of GDM between the phenotypes of PCOS.	The prevalence of GDM is consistent between the phenotypes of PCOS.
33	Dehghani Firoozabadi et al., 2019 [45]	Iran	Cross-sectional study (n = 200)	Phenotype A was associated with an increased risk of GDM; phenotype B was associated with an increased risk of PIH. Among phenotypes, no significant differences were found in the incidence of neonatal complications.	Findings seen in phenotypes A and B indicate a need for more intensive prenatal care.
34	Kouhkan et al., 2019 [11]	Iran	Retrospective cohort study (n = 270)	The most significant predictors of developing GDM were a history of PCOS, progesterone injections, and previous ovarian hyper-stimulation syndrome risk. Administration of injectable progesterone at the beginning of pregnancy was associated with a two-fold risk increase in the development of GDM when compared to vaginal progesterone.	The risk of GDM development is significantly increased with patients who have a history of PCOS, as well as a history of ovarian hyper-stimulation syndrome and progesterone injections.
35	Aktun et al., 2016 [32]	Turkey	Case-control study (n = 1,360)	GDM had a higher incidence in the PCOS group than the control group. Between both groups, there was a significant difference in the incidence of neonatal hypoglycemia, occurring more than twice as often in the PCOS group.	Among patients with PCOS and GDM, there is an increased risk of preeclampsia and neonatal hypoglycemia.
36	De Wilde et al., 2014 [37]	Netherlands	Prospective cohort study (n = 326)	Among patients with PCOS, GDM, PIH, premature delivery, and infants who were small for gestational age were the most common maternal complications.	GDM was the pregnancy complication that occurred most often in patients with PCOS.
37	De Wilde et al., 2015 [14]	Netherlands	Prospective cohort study (n = 72)	Insulin, testosterone, and SHBG levels were lower in patients with PCOS who did not develop GDM.	Women with PCOS who have disturbed insulin, as evidenced by higher insulin levels at the beginning of gestation, clarify the pathogenesis of GDM in this population of people.
38	De Wilde et al., 2017 [48]	Netherlands	Prospective cohort study (n = 188)	There is a significant risk associated with the development of GDM in patients with hyperandrogenic PCOS, as well as infants who are born small for gestational age in this subgroup of patients.	Hyperandrogenic PCOS is associated with an increased risk of both maternal and neonatal complications.
39	Helseth et al., 2013 [17]	Norway	Randomized control trial (n = 274)	GDM was seen in patients who were of shorter stature and had increased insulin levels. Among patients with GDM, there was reduced total weight gain in pregnancy.	The prevalence of GDM did not differ in the two study groups; however, in patients with GDM, less weight loss in the 1^st^ year postpartum was seen.
40	Joham et al., 2014 [33]	Australia	Cross-sectional study (n = 14,799)	GDM and T2DM were present significantly in patients with PCOS compared to controls.	PCOS was the risk factor associated with increased odds of developing GDM and T2DM.
41	Kakoly et al., 2017 [18]	Australia	Secondary analysis (n = 8,009)	Patients with PCOS who developed GDM were significantly associated with a higher BMI trajectory.	If able to reduce weight in early adulthood, patients with and without PCOS can decrease their risk of GDM during pregnancy.
42	Li et al., 2021 [27]	China	Observational study (n = 196)	In patients with PCOS and GDM, compared to PCOS without GDM, significant differences were found in age, BMI, insulin resistance index, fasting insulin, testosterone, and SHBG levels. Additionally, the incidence of pregnancy and neonatal complications was significantly higher in the PCOS and GDM groups.	Given the significantly higher incidence of GDM in patients with PCOS, it is imperative to target the identified risk factors to reduce the occurrence of GDM.
43	Liu et al., 2021 [20]	China	Retrospective case-control study (n = 204)	Patients with PCOS with a history of early pregnancy loss have a significantly higher rate of cesarean section and risk of GDM development during pregnancy. BMI above 28 in patients with PCOS was associated with an increased incidence of GDM.	In patients with PCOS, a history of early pregnancy loss and obese BMI were factors that increased the risk of GDM; therefore, early intervention in patients who fall within one or both of these categories can improve pregnancy outcomes.
44	Rees et al., 2016 [43]	UK	Retrospective observational study (n = 9,068)	Patients with PCOS exhibited significantly increased odds of miscarriage, GDM, premature delivery, C-section delivery, neonatal jaundice, and neonatal respiratory complications compared to controls.	Irrespective of BMI, patients with PCOS are more prone to adverse pregnancy and neonatal outcomes.
45	Sawada et al., 2015 [23]	Japan	Case-control study (n = 113)	In patients with PCOS, a higher incidence of GDM was observed in the first trimester. Patients with PCOS and obese BMIs had a higher incidence of GDM associated with severe insulin resistance.	Among patients with PCOS, there is an increased risk of GDM, which worsens when patients also have higher BMI and poor insulin secretion.
46	Li et al., 2018 [19]	China	Prospective cohort study (n = 248)	30.2% of women with PCOS developed GDM, and higher body mass, insulin resistance, fasting plasma glucose, elevated blood pressure, free androgen index, abnormal cholesterol, less gestational weight gain, and lower levels of SHBG were found to be risk factors.	There is a high prevalence of GDM in pregnant women with PCOS, and it is associated with numerous metabolic risk factors.
47	Xiao et al., 2016 [42]	China	Retrospective cohort study (n = 2,389)	Patients with PCOS had a significantly higher risk of GDM development during pregnancy and preterm delivery.	Patients with PCOS are more likely to develop GDM and have a preterm birth.
48	Li et al., 2018 [34]	China	Retrospective cohort study (n = 6,000)	Compared to groups A and C, group B has a higher prevalence of pregnancy complications of GDM, PIH, and premature delivery; however, no significant differences in prevalence were found in neonatal outcomes.	Findings suggest that patients with PCOS are at an increased risk for GDM and PIH.

Some limitations to the study surround the lack of research determining phenotype variation and effects of fertilization methods. GDM is multifactorial and can be a dangerous complication of pregnancy, so evaluating many aspects associated with pregnancy is crucial to understanding and preventing the development of GDM. Some studies found hyperandrogenism itself may increase the risk of PCOS. Of the few studies observing phenotypic differences, this was mostly agreed upon, but the lack of research limits this significance. Fertilization should be evaluated more as the actual conception can influence the pregnancy, and it should be evaluated how assisted fertilization impacts pregnancy outcomes.

## Conclusions

Several risk factors increase the risk of GDM in women with PCOS, such as a high BMI, older age, increased free androgen index, insulin resistance, preconception impaired glucose tolerance, decreased SHBG, family history of type 2 diabetes mellitus, and even hypothyroidism. Additional pregnancy complications can occur, with an increase in pregnancy-induced hypertension being the most noticeable. Increased pregnancy complications, in general, were found to be significantly higher in women who had prior amenorrhea compared to oligomenorrhea. Infants were also at an increased risk of being born with a decrease in birth weight and having a difference in weight gain during the 1st year of life. As known, PCOS is positively associated with an increased risk of GDM and, across all studies, has significant prevalence. These women also tend to be overweight with an increase in BMI. Some studies find that phenotype A was more likely to develop GDM than the other phenotypes. When it comes to fertilization, hormone therapy such as clomiphene citrate and FSH showed an increased risk of GDM, but there was an inconsistency with ART as some studies indicated a decreased prevalence. At the same time, others found that IVF may increase the prevalence of GDM.

GDM in the setting of PCOS greatly affects the pregnancy outcome and neonatal complications, and they do vary from patients with either just GDM or just PCOS. As seen, there are some traits of specific outcomes due to this pairing, such as a mother with PCOS and GDM tend to have low birth weight babies, even though GDM is associated with large gestational weight and further complications such as shoulder dystocia. There are a lot of factors involved in the development of this interaction. Because outcomes specifically due to the combination of PCOS and GDM can lead to significant consequences to both the mother and fetus, it is imperative to identify the unified risk factors that increase the chances of preventing this interaction from occurring. Further research should also focus on possibly creating a predictive model concerning predictors and risk factors before or at the beginning of pregnancy to implement treatment protocols to drastically reduce the occurrence of GDM.
